# Person-Centered Health Intervention Programs Provided at Home to Older Adults with Multimorbidities and Their Caregivers: A Systematic Review

**DOI:** 10.3390/healthcare14060815

**Published:** 2026-03-22

**Authors:** Vânia Nascimento, Mauro G. Lopes, Miguel M. Leitão, César Fonseca, Elisabete Alves, Isabel Bico, Lara Guedes de Pinho

**Affiliations:** 1 Nursing Department, Universidade de Évora, 7000-811 Évora, Portugal; cfonseca@uevora.pt (C.F.); elisabete.alves@uevora.pt (E.A.); isabel.bico@uevora.pt (I.B.); 2 Unidade Local de Saúde do Litoral Alentejano, 7540-230 Santiago do Cacém, Portugal; maurolopes22@gmail.com; 3 Instituto de Investigação e Formação Avançada (IFA), Universidade de Évora, 7002-554 Évora, Portugal; 4 Comprenhensive Health Research Centre (CHRC), Universidade de Évora, 7004-516 Évora, Portugal; l.pinho@ess.ipvc.pt; 5 Unidade Local de Saúde de Almada-Seixal, 2805-267 Almada, Portugal; miguelamleitao@gmail.com; 6 Escola Superior de Saúde, Instituto Politécnico de Viana do Castelo, 4900-314 Viana do Castelo, Portugal

**Keywords:** patient-centered care, aged, caregivers, home care services

## Abstract

There is a high rate of morbidity and considerable functional dependence in older adults, requiring care from informal caregivers. Person-centered care is a personalized approach that meets the person’s needs, taking their context into account. **Objectives:** Our aim was to assess the available evidence on Person-Centered Health Intervention Programs (PCHCIPs) in a home setting among older adults with multimorbidity and their informal caregivers, namely regarding their main characteristics and respective health outcomes. **Methods:** A systematic literature review was conducted in accordance with the PRISMA guidelines. Bibliographic searches were performed in five databases (CINAHL, MEDLINE, MedicLatina, Scopus, and Psychology and Behavioral Sciences Collection) in November 2024, including studies published between January 2014 and November 2024. Inclusion criteria: Randomized Controlled Trials of person-centered health intervention programs delivered to older adults and their caregivers in home care settings, and scientific articles published betweeen 2014 and 2024. The review protocol was registered in PROSPERO (registration number: CRD42022303687. **Results:** Twelve articles were included (*n* = 12). The PCHCIPs provides psychoeducational training and empowerment with physical, psychological, and social interventions that result in health outcomes for the dependent older adults (decreased health-related events and increased social involvement), for the caregiver (improved QoL, reduced burden), and for both participants (increased life satisfaction and reduced costs in health). **Conclusions:** PCHCIPs covering different areas of intervention (physical, psychosocial and economic) demonstrate positive health outcomes for older adults and their caregivers. It is important to explore more programs that encompass both participants.

## 1. Introduction

The global population is aging declining mortality and birth rates, which are changing health and disease patterns worldwide [1,2,3]. Progress in the social, public health and economic domains, such as changes in health-related behaviours, improved availability and access to healthcare services, and increased life expectancy, is also contributing to these changes [3,4].

An estimated 2 billion people aged 60 years and older will be living by 2050, according to WHO data, twice the population of approximately 900 million in 2015. It was estimated that this population would exceed one billion by 2020. This demographic transition has major consequences for health systems, primarily driven by an increasing burden of chronic diseases and functional limitations among older people as well as continuing demand for long-term care [5].

Aging is a natural process that every human being experiences uniquely [6,7], implying physical, biological, psychological, and social changes. Older adults, defined as individuals aged 65 or older [8], exhibit a higher prevalence of several chronic diseases, especially when institutionalized [9]; thus, quality aging requires lifelong care [10,11,12].

The functional, sensory, and cognitive alterations, as well as multimorbidity and social isolation [13,14], that frequently coexist among older adults may contribute to greater dependency [9,12,15], which can lead to more complex health and economic challenges [12,16]. Also, functional dependence, disability, and multimorbidity tend to negatively affect the quality of life of older adults [12]. In this review, multimorbidity is understood as the coexistence of two or more chronic conditions in the same individual.

Older adults with multimorbidity frequently present complex and long-term care needs that require coordinated and continuous care approaches. As a result, they often require greater support across different areas of care, which may contribute to fragmented care provision and increased demands on health systems [12,17]. Given these multidimensional care requirements, models that provide coordinated, continuous care in the person’s living environment have become increasingly relevant.

In this context, to positively address declines due to aging, priority should be given to home care [16]. However, as older adults lose functional capacity, they increase their dependence on others, namely, informal caregivers [14].

Home-based care has emerged as a key approach to support older adults with multimorbidity. This facilitates care delivered in the person’s social environment and external circumstances, helping maintain continuity of care while avoiding unnecessary admissions. For individuals with multiple chronic conditions, home-based interventions can facilitate integrated, personalized care that addresses complex health challenges while also enhancing functional independence and quality of life [12,14,16].

The informal caregiver is defined as a relative of a dependent person who can or does not cohabit with the person and does not receive monetary remuneration for the care provided [18,19]. A caregiver provides satisfaction with life activities that the person cannot perform alone, such as feeding, medication management, or hygiene [14,18]. Although informal caregivers may feel fulfilled by this role, the social and economic strains they face may lead to overload and stress [18]. Thus, the physical and mental health of the informal caregiver deserves special attention from health professionals [14,18] and should be assessed from a multilevel approach.

The Institute of Medicine identify Person-centered care as essential for improving the quality of health care in 2001, and the implementation of person-centered strategies and interventions has gained increasing attention in the scientific literature over the past decade [20]. World Health Organization (WHO) [5] defined person-centered care as an essential approach to personalizing care across the different stages of life, warning of the need for a paradigm shift in health care. Person-centered care is care that respects the values, preferences, and needs of the person, promotes informed decision-making, and respects the rights of the person cared for. This care requires an effective, empathetic relationship between the health professional and the person cared for [21].

Also, Pinho et al. [22] emphasized the importance of family involvement, namely in monitoring medication side effects and therapeutic adherence, and in developing a relapse prevention plan. Scientific literature supports the idea that person-centered care increases satisfaction, health outcomes, therapeutic adherence, and, consequently quality of life, while ensuring human and ethical rights in the process [23,24,25].

Multimorbidity in older adults is associated with complex and protracted care needs, deepening reliance on informal caregivers who are already utilized for most of the long-term care. This dynamic not only challenges caregivers but also affects the well-being of both members of the dyad. This reality reinforces the need for care plans tailored to the unique needs, preferences, and circumstances of older adults and their caregivers [10,12].

Person-Centered Health Care Intervention Programs (PCHCIP) focus on citizens’ feelings about the disease [24], prioritizing the efficiency of communication between the person and the health professionals [24], citizens’ empowerment, and informed decision-making [26].

For the purposes of this review, healthcare intervention programmes are identified as person-centered if they incorporate fundamental principles of an explicit person-centered and participatory model of care. The principles include a comprehensive and personalised assessment of needs, collaboration with older people and/or their carers in the care planning process, and strategies to improve empowerment, shared decision-making and self-management [5,20,21].

Although person-centered care is widely recognized as a best practice in health care, its definition and implementation vary widely across settings [27]. Based on this literature, PCHCIPs have shown promise as interventions to improve the outcomes and experiences of care for older adults by using consistent empowerment strategies and interactions with health care professionals [28]. However, the evidence remains fragmented, as interventions are often designed with different clinical focuses that may be pathogen-specific, and many have not integrated informal caregivers into care delivery. Additionally, there has been limited synthesis of the scientific literature on the effectiveness and efficiency of PCHCIPs delivered in home environments, especially those focused on older adults with multimorbidity and their caregivers [27,28].

The aim of this review is to assess the available evidence on Person-Centered Health Intervention Programs (PCHCIPs) in a home setting among older adults with multimorbidity and their informal caregivers, including the main characteristics of these programs and their respective health outcomes.

Specifically, this review aims to:

- Identify the characteristics of person-centered health care intervention programs implemented in home settings.
- Analyse the health outcomes associated with these interventions for older adults and their informal caregivers.
- Examine the social and economic outcomes reported in the included studies.

Accordingly, the review addresses the following research question: “Which person-centered health care intervention programs provided at home exist that bring health outcomes of older adults with multimorbidity and their informal caregivers?”

## 2. Materials and Methods

### 2.1. Eligibility Criteria

Eligibility criteria for study inclusion were as follows: (1) Randomized Controlled Trials (RCTs) published between 2014 and 2024 in Portuguese, English and Spanish; (2) target population aged 65 years or older with multimorbidity, defined as the coexistence of two or more chronic conditions in the same individual; (3) inclusion of informal caregivers of legal age; (4) evaluation of health outcomes both for the dependent older adult and for the informal caregiver; (5) PCHCIP provided in home settings in any geographical area.

### 2.2. Exclusion Criteria

Exclusion criteria for study selection were as follows: (1) studies that did not involve both older adults and informal caregivers; (2) studies not conducted in home care settings; (3) non-randomized or observational studies; (4) conference abstracts, editorials, and opinion papers; (5) studies not written in Portuguese, English, or Spanish.

### 2.3. Data Sources

A comprehensive database search was conducted in CINAHL, MEDLINE, MedicLatina, Scopus, and Psychology and Behavioral Sciences Collection in November 2024, and included studies published between January 2014 and November 2024 to capture the most recent evidence on person-centered health care intervention programs reported in the scientific literature during the last decade.

The search strategy combined Medical Subject Headings (MeSH) terms and keywords related to person-centered care, older adults, home care, caregivers, and randomized controlled trials. The search strategy used in MEDLINE (via EBSCO) was as follows, using a combination of Medical Subject Headings (MeSH) terms and free-text keywords: (patient-centered care OR client-centered care) AND (older adults OR elderly OR aged) AND (home care) AND (caregivers OR Caregiver Burden) AND (Randomized controlled trial).

In CINAHL and MedicLatina, equivalent search terms were used, with controlled vocabulary tailored to the respective indexing systems. In Scopus and Psychology and Behavioral Sciences Collection, searches were conducted using keyword combinations in the TITLE-ABS-KEY fields.

Filters were applied to include studies published between January 2014 and November 2024 and restricted to articles written in English, Portuguese, and Spanish and to randomized controlled trials.

### 2.4. Data Selection

Studies were exported to Rayyan [29], and duplicates were identified and removed. Two reviewers (VN and ML) independently screened studies, initially based on the title, abstract, and keywords, and afterwards, based on the full text. During the full-text screening phase, the study population was evaluated for multimorbidity based on whether the design met eligibility criteria for coexistence (i.e., two or more chronic conditions).

Those who did not meet the inclusion criteria were excluded. A third reviewer (LGdP) was consulted in the event of disagreement or doubt. Disagreements between reviewers were resolved through discussion and consensus, with the third reviewer involved when necessary.

The study selection process followed the Preferred Reporting Items for Systematic Reviews and Meta-Analyses (PRISMA) guidelines.

### 2.5. Data Extraction

During the data extraction phase, a descriptive assessment of each study was conducted using a predefined data extraction tool developed according to the research question. Data on authors and years of publication, country, period of data collection, participants and sample, intervention content, intervention delivery mode, data collection methods, and health outcomes (instruments) were collected.

The two initial reviewers independently performed the data extraction. The third reviewer analysed the studies when there was no agreement between the two initial reviewers.

### 2.6. Quality Appraisal

The assessment of methodological quality and levels of evidence was carried out by two reviewers using the JBI Critical Appraisal Checklist for Randomized Controlled Trials [30].

In the JBI Critical Appraisal Checklist, “Yes” answers were given 1 point, and “No” and “unclear” answers were given 0 points [30].

Each reviewer assigned methodological quality ratings to each of the studies independently, and disagreements between the two were discussed with a third reviewer.

The methodological quality of each article was assessed according to the recommendation of JBI Institute as high quality if the score was above 70%, moderate quality if the score was between 50 and 70% and low quality if the score was below 50% [30]. The levels of evidence were classified according to the JBI recommendations [31].

The protocol of the systematic literature review conducted was previously registered in the International Prospective Register of Systematic Reviews (PROSPERO) under the number CRD42022303687 and published [32]. It was carried out according to the Preferred Reporting Items for Systematic Reviews and Meta-Analyses (PRISMA) to answer the research question.

The protocol for this systematic review was registered in the International Prospective Register of Systematic Reviews (PROSPERO) under the registration number CRD42022303687 and previously published [32]. The review was conducted in accordance with the Preferred Reporting Items for Systematic Reviews and Meta-Analyses (PRISMA) guidelines. During the review, the search period was updated to include studies published through November 2024 and to focus on the most recent decade of evidence (2014–2024). This amendment was made to ensure that the review captured the most recent developments in person-centered health care intervention programs and did not affect the objectives or eligibility criteria of the review.

### 2.7. Data Synthesis

Health outcomes reported in the included studies were extracted and described, including statistical significance (*p*-values) and confidence intervals when available. Additional quantitative information, such as mean differences and effect sizes, was also extracted when reported in the original studies.

Table A2 (Appendix A) presents the results of the narrative synthesis [33]. The table summarizes the participants, type of intervention, data collection methods, instruments used to assess health outcomes, and the main results reported for older adults, caregivers, and dyads (older adults–caregivers). Quantitative results reported in the original trials (e.g., *p*-values, mean differences, or effect sizes) are also included when available.

Due to heterogeneity across the included studies in intervention components, outcome measures, assessment instruments, and follow-up periods, a quantitative meta-analysis was deemed inappropriate. In addition, the studies targeted heterogeneous populations, including older adults with stroke or dementia. Therefore, the findings were synthesized using a narrative synthesis approach.

## 3. Results

**Figure A1:**
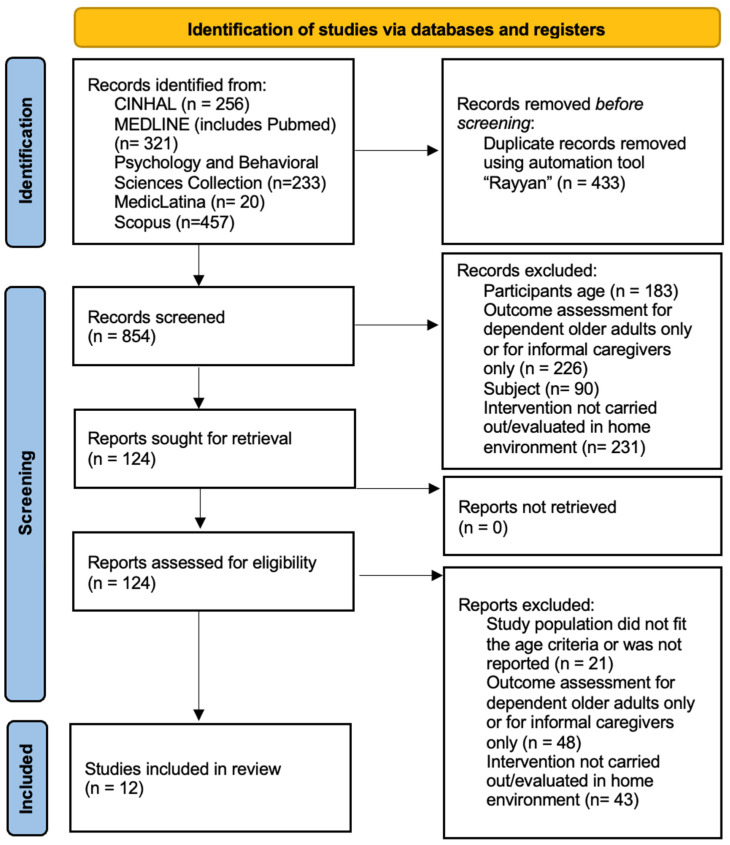
Systematic Literature Review diagram: PRISMA flowchart.

The database search identified 1287 records. After removing duplicates, 854 publications remained for screening. Following the title and abstract screening process, 124 articles were selected for full-text assessment. Of these, 12 studies met the inclusion criteria and were included in the present systematic review (Appendix A, Figure A1).

Table A2 (Appendix A) summarizes the main characteristics of the included studies. The interventions were conducted in different clinical contexts involving older adult–caregiver dyads. Two studies focused on stroke survivors and their informal caregivers [34,35], while seven studies involved older adults with dementia and their caregivers [36,37,38,39,40,41,42]. One study involved older adults with delirium following hospitalization [43], one involved individuals requiring rehabilitation [44], and another included patients with end-stage heart failure [45]. In all studies, informal caregivers were adults, and the older adult participants had a mean age of 65 years or older.

The interventions were directed to different target groups. Two studies implemented interventions exclusively for informal caregivers [35,40], one study focused only on older adults [44], and nine studies implemented interventions targeting both members of the dyad [34,36,37,38,39,41,42,43,45]. Sample sizes ranged from 75 to 996 participants.

The outcomes were assessed at different follow-up periods. One study reported outcomes up to one month after the intervention [43], one between four and twelve weeks [45], two studies up to three months [34,38], two between six and nine months [37,39], one up to twelve months [42], and several studies reported follow-up periods between one and two years after the intervention [35,36,40,41,44].

Across the included studies, multiple outcomes were assessed using validated instruments. Among older adults, the most frequently evaluated domains included functional independence (e.g., Functional Independence Measure—FIM; CAFU), cognition (e.g., Verbal Fluency Test—VF; Clock Drawing Test—CDT; Mini-Mental State Examination—MMSE), subjective memory, symptom severity (e.g., Confusion Assessment Method—CAM; Revised Memory and Behavior Problem Checklist—RMBPC), patient satisfaction, symptom burden (e.g., Edmonton Symptom Assessment Scale), depressive symptoms (e.g., Cornell Scale for Depression in Dementia—CSDD), anxiety (e.g., Hospital Anxiety and Depression Scale—HADS), and social connection.

Among caregivers, the most frequently evaluated outcomes included health-related quality of life (e.g., EQ-5D-3L), overall quality of life (e.g., McGill Quality of Life Questionnaire), caregiver burden (e.g., Zarit Burden Interview—ZBI; Caregiver Burden Inventory—CBI), caregiver strain (e.g., Caregiver Strain Index—CSI), perceived well-being, stress (e.g., Perceived Stress Scale—PSS), and positive caregiving experience.

Some studies also assessed outcomes at the dyadic level, including life satisfaction (e.g., Satisfaction with Life Scale—SWLS [46]), satisfaction with care, depressive mood, healthcare costs, and use of health services.

### Comparative Analysis of Intervention Approaches

The interventions assessed in this review fall into two general categories: caregiver-focused interventions and dyadic interventions involving both the older adult and the caregiver. Several interventions included mixed or multicomponent approaches combining caregiver education, psychosocial support, and functional rehabilitation strategies. Specifically, two studies focused mainly on caregivers, and another provided both caregiver-only and dyadic intervention arms, with significant improvements predominantly noted in caregiver burden and psychological well-being. Most trials that delivered dyadic interventions reported outcomes related to functional independence, cognitive performance, quality of life, and caregiver well-being among older adults and caregivers. However, given intervention types, outcome measures, and follow-up durations, a standard quantitative comparison was not possible.

## 4. Discussion

The studies from this review suggest that PCHCIPs emphasize family inclusion and empower the older adult–caregiver dyad. These programs combine psychosocial interventions with functional rehabilitation strategies and are associated with improvements in health and caregiving outcomes.

Several studies reported reductions in healthcare service use or related costs following the implementation of person-centered interventions [36,40,44]. For example, Brusco et al. [44] found fewer hospital readmissions and lower service use in rehabilitation programs. Laakkonen et al. [36] and Birkenhäger-Gillesse et al. [40] reported reductions in healthcare and social care costs from multicomponent interventions for older adults with dementia and their caregivers. These results suggest that such interventions may improve healthcare resource efficiency, particularly by reducing hospital readmissions and service use [36,40,44]. Functional improvements and enhanced autonomy in older adults may drive these effects, thereby reducing the need for informal caregiving [39,44].

Previous systematic reviews and meta-analyses have reported similar conclusions [47,48,49]. Doungsong et al. [42] also found that multidisciplinary exercise programs may reduce healthcare resource use.

Caregiver empowerment emerged as a central component of many programs and improved outcomes for both caregivers and dependent older adults [34,35]. Education, skill-building, and behavioral management strategies enhance caregivers’ confidence and coping skills while strengthening caregiving relationships [34,35,38,39]. Training and empowerment are key elements of person-centered care, supporting active participation by individuals and families in care processes [50]. Chen et al. [51] also noted that caregivers’ coping abilities vary, highlighting the need for interventions tailored to each dyad.

Person-centered approaches may improve outcomes for both members of the dyad. For example, Ng [45] found that home-based palliative care improves quality of life and symptom control in older adults while reducing caregiver stress. Similar benefits have also been reported in cases where older adults do not qualify for palliative care [52].

Strategies promoting caregiver empowerment may increase life satisfaction among caregivers and older adults [34,35]. Mei et al. [34] reported that interventions targeting both the older adult and the caregiver are more effective than those targeting only the caregiver. Interventions that strengthen caregivers’ behavioral management skills have also been associated with reduced depressive symptoms and improved caregiving experiences [39,53].

Several studies emphasize that older adult–caregiver dyads have complex needs that require tailored care strategies. Multicomponent programs may include health education, psychoeducational approaches, coping and stress-management strategies, and psychosocial rehabilitation models that support self-management skills [36,37]. These programs are typically delivered by multidisciplinary teams involving healthcare professionals, family members, and community support services.

Innovative approaches have also been described. Cheung et al. [41] reported that home-based music-guided movement programs may promote psychosocial well-being for older adults and caregivers. Other studies of music-based approaches report similar improvements in physical, psychological, and behavioral outcomes [54,55,56]. Doungsong et al. [42] also found that face-to-face exercise programs may outperform technology-mediated approaches.

Other dyadic strategies focusing on empowerment and relationship dynamics may be particularly relevant for complex older adult–caregiver relationships. Yu et al. [38] reported improvements in cognitive function, subjective memory, and mood among older adults. These findings align with studies suggesting that caregiver education programs may benefit cognitive outcomes in older populations [35,36].

Moreover, person-centered approaches that incorporate psychological support for caregivers may offer important benefits. Improvements in caregivers’ physical and mental health may enhance their ability to continue providing care over time [36,37,38,39,57,58,59]. Several studies also reported improvements in caregivers’ health-related quality of life and caregiving experiences [34,35,38,45]. Psychoeducational programs that include skill-building components may further strengthen caregivers’ coping abilities and confidence [60].

## 5. Implications for Practice, Policy, and Research

From a practical perspective, home-based person-centered care targeting the older adult–caregiver dyad may improve health outcomes and enable a more efficient use of healthcare resources. Home care services may benefit from integrating structured caregiver education and skills training into person-centered programs, strengthening caregivers’ competencies while promoting independence among older adults.

Several studies reported that caregiver education and empowerment are key components of effective home-based programs. They contribute to reductions in caregiver burden, improvements in psychological well-being, and enhanced caregiving competence [34,35,38,39,40]. However, these economic benefits were not consistently observed across all included RCTs and were mainly reported in specific contexts, such as multicomponent interventions in dementia care or rehabilitation settings [36,40,44].

Programs emphasizing caregiver education, skills training, and personalized care planning appear particularly effective in promoting functional independence among older adults. Programs combining functional rehabilitation, psychosocial support, and self-management guidance may help caregivers provide effective home care and support ageing in place.

### 5.1. Limitations of the Studies Included

Most studies reviewed were of moderate to high methodological quality, although several potential biases were identified. Psychosocial interventions were implemented in home settings, making it difficult to blind participants and intervention providers. In addition, several studies reported inadequate blinding of outcome assessors.

Attrition and loss to follow-up were reported in several trials, particularly among frail older adults or those with progressive diseases. Some outcomes relied on self-reported measures, which may introduce recall bias. Other limitations included relatively small sample sizes, the inclusion of motivated volunteers, and interventions delivered within specific healthcare systems, which may limit the generalizability of the findings.

There was substantial heterogeneity across studies in terms of populations, interventions, follow-up periods, and outcome measures. Most studies focused on older adults with dementia or cognitive impairment, and definitions of multimorbidity varied widely. These factors reduced the comparability of findings and led to the decision not to conduct a meta-analysis. Although a comprehensive search strategy was implemented, publication bias cannot be ruled out.

Several interventions were implemented across different clinical contexts, including delirium prevention at hospital discharge, home palliative care for patients with heart failure, and rehabilitation programs to improving post-hospitalization outcomes. These findings suggest that core elements of person-centered dyadic programs may also be relevant for other chronic conditions, such as cardiometabolic diseases. However, further research is needed to confirm their effectiveness in populations with predominantly non-cognitive multimorbidity.

### 5.2. Future Research Directions

Future studies should focus on developing and evaluating person-centered interventions for older adult–caregiver dyads in home environments. Attention should be given to a broader range of clinical conditions and demographic groups. Larger and longer-duration randomized controlled trials are needed to strengthen the evidence base. Future research should also assess the economic and operational feasibility of these programs across different healthcare systems and community care settings.

## 6. Conclusions

This systematic review identified person-centered health care intervention programs implemented in home settings targeting older adult–caregiver dyads. The findings indicate that these interventions may improve functional independence, cognitive outcomes, quality of life, and caregiver well-being. Some studies also reported reductions in healthcare service utilization following the implementation of these interventions.

These findings support the relevance of person-centered approaches that actively involve both older adults and informal caregivers in the care process.

From a clinical perspective, implementing person-centered home-based interventions may improve care quality, strengthen caregiver support, and promote greater autonomy and well-being among older adults.

## Data Availability

No new data were created or analyzed in this study. All data supporting the findings of this systematic review are available in the referenced published articles.

## Abbreviations

The following abbreviations are used in this manuscript:

PCHCIPs	Person-Centered Health Intervention Programs
QoL	Quality of Life
WHO	World Health Organization
RCTs	Randomized Controlled Trials

## Appendix A

**Table A1:** Quality assessment of the included studies.

Checklist														JBIQS	LE
Reference	Q1	Q2	Q3	Q4	Q5	Q6	Q7	Q8	Q9	Q10	Q11	Q12	Q13		
Brusco et al. [44]	U	U	Y	N	N	Y	Y	Y	Y	Y	Y	Y	Y	69%	1.c
Laakkonen et al. [36]	Y	Y	Y	Y	N	U	Y	Y	U	Y	Y	Y	Y	77%	1.c
Verloo et al. [43]	Y	Y	Y	Y	N	Y	Y	Y	U	Y	Y	Y	Y	85%	1.c
Berwig et al. [37]	Y	Y	Y	Y	N	Y	Y	Y	U	Y	Y	Y	Y	85%	1.c
Ng et al. [45]	Y	Y	Y	U	N	Y	Y	Y	Y	Y	Y	Y	Y	85%	1.c
Mei et al. [34]	Y	Y	Y	U	N	Y	Y	Y	U	Y	Y	Y	Y	77%	1.c
Yu et al. [38]	Y	Y	Y	U	N	N	Y	Y	U	Y	Y	Y	Y	69%	1.c
Gitlin et al. [39]	Y	Y	Y	N	N	Y	Y	Y	Y	Y	Y	Y	Y	85%	1.c
Birkenhäger-Gillesse et al. [40]	U	U	Y	U	U	U	Y	Y	Y	Y	Y	Y	Y	62%	1.c
Kang & Li [35]	Y	Y	Y	Y	U	U	Y	Y	Y	Y	Y	Y	Y	85%	1.c
Cheung et al. [41]	Y	Y	Y	N	N	Y	Y	Y	Y	Y	Y	Y	Y	85%	1.c
Doungsong et al. [42]	Y	Y	Y	N	N	Y	N	Y	Y	Y	U	Y	Y	69%	1.c
Y: Yes; N: No; U: Unclear; JBIQS: JBI quality score; LE: Level of evidence															
The questions corresponding to Q1–Q13 of the JBI critical appraisal checklist:															
Question	Description														
Q1	Was true randomization used for assignment of participants to treatment groups?														
Q2	Was allocation to treatment groups concealed?														
Q3	Were treatment groups similar at baseline?														
Q4	Were participants blind to treatment assignment?														
Q5	Were those delivering treatment blind to treatment assignment?														
Q6	Were outcome assessors blind to treatment assignment?														
Q7	Were treatment groups treated identically other than the intervention of interest?														
Q8	Was follow-up complete and, if not, were differences between groups described and analyzed?														
Q9	Were participants analyzed in the groups to which they were randomized?														
Q10	Were outcomes measured in the same way for treatment groups?														
Q11	Were outcomes measured in a reliable way?														
Q12	Was appropriate statistical analysis used?														
Q13	Was the trial design appropriate?														

**Table A2:** Main characteristics of the 12 studies included in the systematic review.

Authors, Year	Country	Period of Data Collection	Participants and Sample	Intervention Content	Intervention Delivery Mode	Data Collection Methods	Health Outcomes (Instruments)	Main Results	Quantitative Results
Brusco et al. [44], 2015	Australia	July 2010–June 2011	Older Adults in a rehabilitation program and their informal caregivers Sample size: *n* = 996	Weekend rehabilitation added to usual care	Randomized controlled trial: Intervention group (*n* = 496) Control group (*n* = 500)	Baseline: 6 and 12 months follow-up	Older adults: Functional independence FIM); Health-related quality of life (EQ-5D-3L) Dyad: Cost-effectiveness	Improved functional independence and HRQoL; reduced health service utilization, informal care and hospital readmissions	Between-group FIM difference: 2.0 at 6 months (95% CI 0.0–4.0; *p* = 0.05); 1.3 at 12 months (95% CI −0.9–3.5; *p* = 0.24)
Laakkonen et al. [36], 2016	Finland	September 2011–March 2012	Adults with dementia, and their informal caregivers Sample size: *n* = 136	Self-Management Groups for People with Dementia and Spouses	Randomized controlled trial: Intervention (*n* = 67) Usual care (*n* = 69)	Baseline; 3, 9 and 24 months follow-up.	Older adults: Cognitive function (VF; CDT) Caregivers: Care management ability (SCQ) Dyad: HRQoL (RAND-36; 15D); health and social care costs	Improved cognitive performance; improved spouse physical HRQoL; reduced healthcare costs.	Spouse physical HRQoL change: 1.0 vs. −2.0 (95% CI −0.5–2.5 vs. −3.5–−0.5); *p* = 0.006
Verloo et al. [43], 2016	Switzerland	February–November 2012	Older adults with delirium symptoms and their informal caregivers. Sample size: *n* = 103	Usual homecare and 5 additional nursing patient-centered intervention	Randomized controlled trial Intervention (*n* = 51) Control (*n* = 52)	Baseline and 1-month follow-up	Older adults: Delirium symptoms (CAM); cognitive status (MMSE); functional status (ADL/IADL) Caregivers: Satisfaction with intervention	Reduced delirium symptoms; high feasibility and satisfaction with interventions	No quantitative between-group estimates (means, differences, or 95% CI) explicitly reported; results mainly addressed feasibility outcomes.
Berwig et al. [37], 2017	Germany	Not reported	Older adults with dementia, and their informal caregivers, Sample size *n* = 92	Multicomponent intervention (DE-REACH)	Randomized controlled trial Intervention (*n* = 47) Control (*n* = 45)	Baseline; 6 and 9 months follow-up	Older adults: Cognitive ability (Structured Interview for Dementia) Caregivers: Reaction to challenging behaviors (RMBPC); Caregiver Burden (ZBI); mental health (PHQ); HRQoL; Perceived social support (ESSI)	Reduced caregiver burden, and somatization; improved HRQoL; reduced reaction to challenging behaviours	Caregiver burden reduction: effect size d = 0.91 (post-intervention); moderate effect at follow-up; psychological HRQoL improvement (*p* = 0.012).
Ng et al. [45], 2018	China	May 2013–June 2015	Older adults with end-stage heart failure (ESHF) and their informal caregivers. Sample size: *n* = 84	Home based palliative Heart Failure program using a transitional care framework.	Randomized controlled trial Intervention (*n* = 43) Control (*n* = 41)	Baseline; 4 and 12 weeks follow-up	Older adults: Quality of life (McGill QOL); Palliative Symptoms (ESAS) Caregivers: Caregiver burden (ZBI) Older Adults and caregivers: Satisfaction with care (Patient Satisfaction Questionnaire)	Improved quality of life (physical, psychosocial, existential aspects, and symptom control (dyspnea, emotional function, mastery); increased satisfaction with care; reduced caregiver burden.	Between-group difference in total QoL: *p* = 0.016; caregiver burden reduction at 12 weeks: *p* = 0.024; physical QoL baseline 4.87 (95% CI 4.42–5.32) vs. 4.32 (95% CI 3.84–4.80)
Mei et al. [34], 2018	China	Not reported	Older adults, Stroke survivors and their informal caregivers/spouses (*n* = 75)	Eight-week modified reminiscence therapy	Randomized controlled trial with three groups (1) couples (both participate in the intervention) (*n* = 25); (2) couples (only the caregivers participate in the experimental intervention) (*n* = 22); (3) control group (*n* = 28).	Baseline and 3-month follow-up	Caregivers: Caregiver burden (CBI); positive caregiving experience (PAC) Dyad: Life Satisfaction (SWLS)	Reduced caregiver burden; improved life satisfaction for both caregivers and older adults	Caregiver burden and life satisfaction improved (*p* < 0.001).
Yu et al. [38], 2019	China	February 2018–January 2019	Older adults with mild cognitive impairment and their caregivers. Sample size: *n* = 103	14-week dyadic strength-based empowerment program (D-StEP-MCI)	Randomized controlled trial Intervention (*n* = 52) Control (*n* = 51)	Baseline; post-intervention; 3-month follow-up	Older adults: Cognitive function (MMSE); subjective memory (MIC); depressive symptoms (CES-D-10) Caregivers: Behavioral symptoms and caregiver distress (RMBPC); depressive symptoms (CES-D-10)	Improved cognitive function and subjective memory; reduced symptom severity, caregiver burden and depressive mood	Subjective memory improvement: *p* = 0.007; symptom severity reduction: *p* = 0.001; caregiver depressive mood reduction: *p* = 0.014.
Gitlin et al. [39], 2021	USA	2012–2016	Older Adults with dementia and their caregivers Sample size: *n* = 250	Tailored activity program (TAP)	Randomized controlled trial Intervention (*n* = 124) Control (*n* = 126)	Baseline; 3 and 6 months follow-up	Older adults: Functional dependence ADL/IADL (CAFU) Caregivers: Well-being (13-item PCBI); depression (PHQ-9) Dyad: Health-related events (death, hospitalization, depression/suicidal ideation)	Reduced assistance required in ADLs and IADLs; improved caregiver well-being; reduced health-related events.	IADL assistance reduction: *p* = 0.02 (d = −0.33); ADL assistance reduction: *p* = 0.04 (d = −0.30); caregiver wellbeing improvement: *p* = 0.01 (d = 0.39)
Birkenhäger-Gillesse et al. [40], 2022	The Netherlands	2016–2018	Older adults with dementia and their caregivers. Sample size: *n* = 109	“More at Home with Dementia” intervention, a multicomponent training program	Randomized controlled trial Intervention (*n* = 59) Control (*n* = 50)	Baseline; 6, 12 and 24 months follow-up	Dyad: Quality-adjusted life years (EuroQol-5); health and social care costs	Reduced use of healthcare and formal social care services	Formal social care cost reduction −€14,008 (95% CI €5714–€22,299; *p* = 0.001); healthcare costs −€3167 (95% CI €480–€5855; *p* = 0.02).
Kang & Li [35], 2022	China	September 2018–December 2019	Older adults Stroke survivors and their caregivers Sample size: *n* = 170	WeChat-based caregiver education (WBCE)	Randomized controlled trial Intervention (*n* = 86) Control (*n* = 84)	Baseline; 3, 6, 9 and 12 months follow-up	Older adults: Cognitive function (MMSE); anxiety and depression (HADS) Dyad: Satisfaction with care (Patient Satisfaction score)	Reduced cognitive impairment, anxiety and depression; increased satisfaction among patients and caregivers	Between-group MMSE difference: 27.1 ± 1.8 vs. 26.5 ± 1.5 (*p* = 0.015); cognitive impairment: 30.2% vs. 45.2% (*p* = 0.043)
Cheung et al. [41], 2022	China	May 2014–August 2016 and January 2018–June 2019	Older adults with dementia and their caregivers. Sample size: *n* = 100	12-week Music-with-Movement dyadic intervention	Randomized controlled trial Intervention (*n* = 55) Control (*n* = 45)	Baseline and 12-month follow-up.	Older adults: Depressive symptoms (CSDD) Caregivers: Stress (PSS); positive caregiving experience (PAC) Dyad: Relationship quality (QCCRR)	Reduced depressive symptoms in older adults; reduced caregiver stress; improved positive caregiving experience.	Reduced depressive symptoms, caregiver stress, and improved positive caregiving experience (all *p* < 0.05).
Doungsong et al. [42], 2024	UK	October 2018–September 2022	Older adults with dementia and their caregivers Sample size: *n* = 205	PrAISED promoting activity, independence, and stability in early dementia	Multicenter randomized controlled trial Intervention (before (*n* = 32) and after (*n* = 151) March 2020 (*n* = 95)) Control (before (*n* = 32) and after (*n* = 150) March 2020)	Baseline and 12-month follow-up	Older adults: Fear of falling (FES-I); HRQoL (EQ5D-5L); Caregivers: Carer strain (CSI), Dyad: Social connection (adapted CAQ); healthcare resource use (adapted CSRI)	Improved HRQoL and social connection; reduced fear of falling, caregiver strain and health service use	SROI £0.58–£2.33 per £1 invested (in-person programme).
